# Associations of Social Network- and Individual-Level Factors with HIV Testing, Condom Use, and Interest in PrEP Among Young Black Women

**DOI:** 10.1007/s10508-022-02306-7

**Published:** 2022-06-08

**Authors:** Jaih B. Craddock, Nancy D. Franke, Caroline Kingori

**Affiliations:** 1 School of Social Work, University of Maryland Baltimore, 525 W. Redwood St., Baltimore, MD 20201 USA; 2 College of Health Sciences and Professions, Ohio University, Athens, OH USA

**Keywords:** HIV prevention, Young Black women, Social network, PrEP

## Abstract

To achieve the 2030 goal of ending the HIV epidemic, we must consider social network- along with individual-level factors related to HIV prevention among young Black women (YBW). This cross-sectional study examined egocentric social network- and individual-level data of 180 YBW aged 18–24. Multivariable logistic regression models were used to study social network characteristics and individual sexual behaviors related to HIV prevention behaviors (e.g., HIV testing, condom use, and interest in preexposure prophylaxis, or PrEP). On average, YBW nominated 11 social network members (SNMs; seven friends, two family members, and one sex partner). About 92% of YBW spoke to at least one SNM about condom use and 58% spoke to at least one SNM about HIV testing. Respondents who spoke to a sex partner about condom use had 70% lower odds of being interested in PrEP, but 2.99 times the odds of reporting condom use during last sex. Odds of being tested for HIV in the prior 3 months were significantly increased by 3.97 times for those who spoke to at least one sex partner about HIV testing. However, odds of being interested in PrEP were significantly decreased by 63% for YBW who were tested for HIV in the prior 3 months. Findings underscore that understanding network- and individual-level factors is crucial in increasing HIV testing, condom use, and interest in PrEP among YBW.

## Introduction

Black American women are disproportionally affected by HIV and account for 57% of new HIV diagnoses among women in the USA (Centers for Disease Control and Prevention [CDC], 2020). In 2010, the HIV Care Continuum Initiative was developed to accelerate HIV testing, care, and treatment. Progress in linkage to care, retention in care, medication, and viral suppression has improved over time among Black women, but the same progress is not evident among Black youth younger than 25 years old (Whiteside et al., 2014). Sexual health communication with social network members (SNMs) such as peers, family members, and sex partners is an important factor in HIV prevention (Cederbaum et al., 2017; Craddock et al., 2016; Holloway et al., 2015). Although social network members and social network dynamics are associated with HIV-related behaviors, HIV prevention research targeting young Black women (YBW) focuses largely on individual-level behaviors (e.g., personal choice, self-efficacy and condom use negotiation; Jemmott et al., 2007). Prevention research must also consider the context (e.g., social networks) in which HIV prevention decisions and behaviors are made.

### HIV Prevention Behavior

Condom use, HIV testing, and use of preexposure prophylaxis (PrEP) are key HIV prevention strategies to help meet the goal of ending the HIV epidemic by 2030 (HIV.gov, 2020). Most HIV behavioral interventions to date are aimed at increasing condom use negotiation skills (Boekeloo et al., 2015; Fogarty et al., 2001; Wenzel et al., 2016) and HIV testing behaviors among various populations (Diallo et al., 2010; Jiwatram-Negrón & El-Bassel, 2014; Maulsby et al., 2013). Despite barriers to HIV testing like stigma, negative reactions, fear, negative assumptions, and privacy concerns (Cheong et al., 2018; De Jesus et al., 2015; McDougall et al., 2016), YBW have reported high HIV testing rates (Craddock, 2020; McElrath et al., 2017; Moore & Belgrave, 2019). Yet consistent condom use among YBW continues to remain low (Craddock, 2020). To better understand the inconsistent utilization of HIV prevention strategies among YBW compared to other racial and ethnic groups, it is important to recognize the multifaceted nature of influences in the psychosocial, sociostructural, and individual contexts (Brawner, 2014; Taggart et al., 2020). At the sociostructural level, YBW may experience lack of youth-friendly clinics, lack of sexual health knowledge and resources within their social networks, racism, discrimination, and poverty (Brawner, 2014; LeBlanc et al., 2014; McElrath et al., 2017). Simultaneously, at the psychosocial and individual levels, YBW may experience condom negotiation, sexual coercion, parent–adolescent communication, and misperceptions about HIV transmission (Claxton & van Dulmen, 2013; Taggart et al., 2020; Willie et al., 2017). All these factors interact to determine adequate access and the extent to which YBW successfully engage in HIV prevention behaviors.

PrEP, a biomedical prescription medication that can help decrease the risk of acquiring HIV (CDC, 2018), is a relatively new HIV prevention method that can be tailored for YBW. Research shows that only about one third of Black women had previously heard of PrEP, but once introduced, interest in PrEP increased to between 28% (Hirschhorn et al., 2020) and 63% (Chandler et al., 2020). Additionally, a positive association between self-perceived HIV risk and interest in PrEP has been shown (Park et al., 2019; Sales & Sheth, 2019; Sewell et al., 2020). At the psychosocial level, the influence of relationship dynamics in use of PrEP among YBW is key to counteracting or creating barriers to HIV prevention (Willie et al., 2018, 2019). More research on sexual health communication among sex partners is needed to provide contextually relevant social network interventions among YBW.

The examination of egocentric social networks has been successfully used to understand and reduce HIV risk behaviors in priority populations and their networks (Amirkhanian, 2014; Cederbaum et al., 2017; Craddock et al., 2016, 2020; Holloway et al., 2015; Sheehan et al., 2019; Widman et al., 2014). Egocentric social networks focus on individuals and their social interactions with SNMs who are directly linked to these individuals (Perry et al., 2018). The idea is that each individual (YBW) lives in a personal community of SNMs and the characteristics (e.g., relationships, interactions, communication) of their SNMs are salient in influencing the YBW’s HIV prevention behaviors (Perry et al., 2018; Valente, 2010).

Research examining men who have sex with men, homeless youth, and other priority populations has indicated that sexual health communication with SNMs is associated with HIV prevention behaviors (Amirkhanian, 2014; Cederbaum et al., 2017; Craddock et al., 2020; Holloway et al., 2015; Widman et al., 2014). However, very few studies have examined sexual health communication between YBW and their SNMs. One of the few studies that has done so focused on with which SNMs and how YBW communicate about condom use and HIV testing behaviors (Craddock, 2020). Although this information is important for HIV intervention development, Craddock (2020) did not examine how sexual health communication with SNMs was associated with HIV prevention behaviors (i.e., condom use, HIV testing, and interest in PrEP use). Thus, it is not only necessary to gain an understanding of whom YBW aged 18 to 24 speak to about condom use and HIV testing, but also critical to understand how these conversations are associated with actual condom use, HIV testing behaviors, and interest in PrEP.

It is widely known that YBW do not participate in riskier sexual behaviors than their non-Black counterparts. Nonetheless, several individual-level factors have been associated with higher risk of HIV (e.g., sex under the influence of drugs and alcohol, concurrent sexual partnerships, and informal exchange of sex for resources like money, housing, food, and clothing; Caldwell & Mathews, 2015; Hall & Tanner, 2016; Hutton et al., 2015; Lewis et al., 2015; Morris et al., 2009; Newsome & Airhihenbuwa, 2013). Accordingly, these individual-level factors must be considered alongside social network factors when determining what is associated with condom use, HIV testing, and interest in PrEP among YBW. This study examined how individual-level factors (e.g., concurrent sex partners, sex under the influence) and SNM-level factors (e.g., sexual health communication, relationship type) were associated with condom use, HIV testing, and interest in use of PrEP among YBW.

## Method

### Participants

Egocentric social network data were collected from 200 YBW aged 18 to 24 from June to December 2018. Respondent-driven sampling (RDS) was used to connect with this hard-to-reach and hard-to-engage population. RDS assumes that “those best able to access members of hidden [or hard-to-reach] populations are their own peers” (Heckathorn, 1997, p. 178). RDS involves a dual incentive system (i.e., incentives for participation and referrals) and uses both monetary and symbolic (excitement about contributing to potential solutions for an important problem) rewards (Heckathorn, 1997). A text-enhanced version of RDS (the use of text messages) was used to recruit participants and was effective in recruiting YBW in a short period of time compared to traditional RDS recruitment outcomes (paper tickets; redacted for review).

Fourteen initial participants were recruited via community-based organizations, Facebook, and Twitter. To qualify as a participant, women had to (1) be 18–24 years of age, (2) identify as a Black or African American woman (inclusive of transgender women and any sexual orientation), and (3) ever be sexually active. As part of the RDS process, participants were asked to invite at least three eligible YBW (i.e., friends, family, or acquaintances) to participate in the study, until a maximum of 200 women had completed the study. To qualify as an invited participant, YBW had to meet the same eligibility criteria as initial participants and be invited by a current study participant. Participating YBW were not informed of which invited women took part in the study to ensure confidentiality. Participants received a $10 incentive for inviting other YBW to participate in the study, regardless of if those women participated. Although this study was inclusive of all individuals who identified as women, including transgender women, there were no participants who reported identifying as transgender in this study.

To achieve the study’s aims, social network- and individual-level data were collected from participants via an online self-administered survey. The individual-level portion of the survey included topics related to personal demographics, HIV risk and prevention behaviors, and interest in PrEP. Once the individual-level portion of the survey was completed, participants were prompted to start the social network-level portion of the survey.

To collect egocentric social network data (Matzat & Snijders, 2010), each participant was asked to list the names of their SNMs using a name generator: “Thinking about the last month, list all the people you have communicated with.” Participants listed between five and 20 names of people they had spoken with during the last 30 days. Once participants finished listing the names of their SNMs, each SNM name was turned into an answer option, similar to categorical answers to a multiple-choice question. Questions were then asked about the types of relationships and communication participants had with each SNM they mentioned (e.g., “Who do you talk to about HIV?”). For each question, participants received the names they listed in the name generator and could select all the names relevant to the question. The self-administered survey took approximately 45 min to complete, and participants received $15 for their participation. Study procedures were approved by the [redacted for review] institutional review board.

### Measures

#### Sociodemographic Characteristics

Sociodemographic variables included in this analysis were age (continuous) and level of education (high school diploma or GED [reference group], some college or two-year degree, bachelor’s degree, professional degree). Due to the small number of YBW with professional degrees (*n* = 8), a new variable of bachelor’s degree or more was created to include in the models.

#### Individual-Level Sexual Behaviors

Sexual behaviors were assessed by asking questions about sexual activity and sexual behaviors. Sexual activity was assessed by asking “Have you ever had vaginal or anal sex?” (1 = *yes*, 0 = *no*) and “When was the last time you had sex (oral, vaginal, or anal)?” Responses were dichotomized as 1 (*within the previous 4 weeks*) or 0 (*more than 4 weeks ago*) to create the variable of sex in the prior 30 days. Condom use was measured by asking, “The last time you had sex, what kinds of sex did you have?” (1 = *anal sex with a condom*, 2 = *anal sex with no condom*, 3 = *oral sex with a condom or dental dam*, 4 = *oral sex without a condom or dental dam*, 5 = *vaginal sex with a condom*, 6 = *vaginal sex with no condom*). Responses were dichotomized as 1 (*condom use*) or 0 (*no condom use*). Oral sex was excluded from analyses due to research indicating that the risk of acquiring HIV is low when participating in oral sex (CDC, 2016). Use of hormonal birth control was assessed via the following question: “The last time you had vaginal sex, what method(s) did you or your partner use to prevent pregnancy? Check all that apply.” (1 = *I have never has vaginal sex*; 2 = *no method was used to prevent pregnancy*; 3 *birth control pills*; 4 = *condoms*; 5 = *Depo-Provera (or any injectable birth control), Nuva Ring (or any birth control ring), Implanon (or any implant)*; 6 = *pulling out/withdrawal*; 7 = *some other method*; 8 = *not sure*). Responses were dichotomized as 1 (*birth control pills or any injectable birth control, any birth control ring, any implant*) or 0 (*none or other types of birth control methods*). All women examined in this study reported having vaginal sex.

The following sexual risk variables were assessed in a yes-or-no format (1 = *yes*, 0 = *no*) unless otherwise specified: (1) concurrent sex partners: “In the past 12 months, did you ever have sex (vaginal or anal sex) with one partner, sex with a different partner, and then sex with the first partner again, within a week?”; (2) exchange sex: “Have you ever exchanged sex (oral, vaginal, or anal) for money, a place to stay, food or meals, or anything else?”; (3) sex under the influence: “Did you drink alcohol or use drugs before you had sex (vaginal or anal sex) the last time?”; (4) sex with someone met online: “Have you ever had sex (vaginal or anal) with someone you met online?”; (5) STI testing: “ Have you ever been tested for a sexually transmitted infection, or STI or STD, for example, chlamydia, gonorrhea, syphilis, genital warts?”; (6) ever tested positive for a STI: “Did you test positive for any STDs?”; (7) Tested for HIV in prior 3 months: “When was the last time you were tested for HIV/AIDS?” (1 = *within the past 3 months*, 0 = *3 months or more*); (8) heard of PrEP: “Have you ever heard of the HIV prevention pill called PrEP?; and (9) interest in PrEP use: “Does PrEP sound like something you would be interested in taking to help prevent you from getting HIV?” Additionally, participants were asked if they had ever attended an HIV education program: “Have you ever participated in a HIV/STI educational or prevention program outside of school?”

These questions were derived from the lifetime sex risk questions from the CDC’s Youth Risk Behavior Survey and have been rigorously tested for reliability and validity (Brener et al., 2002). Additional sexual behavior questions were based on those used by Rice and colleagues in prior studies (Rice, 2010; Rice et al., 2010; Wenzel et al., 2016).

#### Social Network Variables

##### SNM Relationships

To assess which SNMs were sex partners, participants were asked “Who have you ever had sex (anal, vaginal, or oral sex) with?” Any SNM listed as a someone they had sex with was recoded as a new dichotomous sex partner variable (1 = *selecting an SNM* and 0 = *no selection)*. To assess which SNMs were friends, participants were asked “Who on this list would you call a friend?” The names of all nominated network members were listed as answer options, and the participant could select individuals corresponding to that question. Any SNM listed as a friend was recoded as a new dichotomous friend variable (1 = *selecting an SNM* and 0 = *no selection)*. To assess which SNMs were family members, participants were asked “What is your relationships with this individual?” Any SNM listed as a family member was recoded as a new dichotomous family member variable (1 = *selecting an SNM* and 0 = *no selection)*.

##### Sexual Health Communication with SNMs

Unidirectional communication questions were combined to assess bidirectional sexual health communication among YBW and their SNMs. The variable for talking about condoms was created using the following questions: “Who have you ever talked to about condoms, or practicing safer sex?” and “Who has ever talked to you about condoms, or practicing safer sex?” (1 = *selecting an SNM* and 0 = *no selection*). The variable for talking about HIV testing was created using the following questions: “Who have you ever talked to about getting an HIV test?” and “Who has ever talked to you about getting an HIV test?” (1 = *selecting an SNM* and 0 = *no selection*).

Interaction variables of talking about condom use or HIV testing with a family member, friend, or sex partner were created to examine the differences between talking with various types of SNMs about each topic. The interaction variable of talking to a family member about condom use was created if an SNM was listed as a family member and someone with whom the YBW talks about condom use. Similar interaction variables were created for talking with a family member, friends, or sex partners about HIV testing and condom use. These variables were binary (1 = *selecting an SNM* and 0 = *no selection*).

### Statistical Analysis

The aim of this analysis was to investigate how social network dynamics may be associated with HIV testing, condom use, and interest in PrEP use among YBW. Because this study focused on HIV prevention behaviors, the sample in this study was limited to YBW who reported ever having vaginal or anal sex (*n* = 168). With a sample size of 168, this study had 80% statistical power to detect a small to medium effect (OR 2.16–2.99) in a one-tailed logistic regression at a 0.05 significance level.

Egocentric network analysis was conducted using SAS to determine the associations among SNM relationship types (i.e., friend, family member, sex partner); sexual health communication between YBW and SNMs (i.e., about condom use, HIV testing); and HIV testing, condom use, and interest in PrEP. Egocentric network analysis allowed for the inclusion of social network variables in standard statistical models, such as multivariable logistic regression. Independent variables constructed from SNM data were created in SAS and merged with individual-level survey data.

Statistical analysis proceeded in two stages: (1) descriptive statistics of the YBW, their sexual risk factors, their sexual health communication, and social network dynamics were calculated; and (2) multivariable logistic regression modeling was conducted. Multivariable logistic regression models were run with each outcome variable. The outcome variables were treated as dichotomous outcomes and regressed on individual-level measures (i.e., measures that only varied across participants) and social network-level measures (i.e., measures that varied across SNMs). Individual-level measures were created based on standard individual responses to survey items. Two methods were used to assess multicollinearity. First, correlation models were ran with the inclusion of all independent and dependent variables. Variables with a 0.70 or higher was considered highly correlated and had potential for multicollinearity. Second, variance inflation factor (VIF) and tolerance were included in each regression model. In the tolerance analysis, any variable with parameter estimate that fell below a 0.1 was considered a threat of multicollinearity. In the VIF analysis, any variable with a parameter estimate value of 10 or higher was considered a threat of multicollinearity. Results from the correlation models and the VIF and tolerance analyses indicated no multicollinearity among the variables included in these analyses. The variable of exchange sex was excluded from the final models due to low frequency (*n* = 6). All final logistic regression analyses were carried out in SAS 9.4.

## Results

### Descriptive Statistics

#### Sexual Behaviors

Of the 168 YBW, 19.05% reported ever participating in an HIV or STI program or intervention outside of school. Among YBW, 52.98% reported sexual activity in the prior 30 days. Risky sexual behaviors included: (1) having sex with someone they met online (39.90%), (2) having sex under the influence of alcohol or drugs (30.95%), (3) concurrent sex partners (10.71%), and (4) participating in exchange sex (3.57%). Among participants, 73.33% reported having ever been tested for STIs; of those who had been tested, 13.69% had ever tested positive for an STI. YBW were also asked about methods used to prevent pregnancy. Slightly more than half (51.79%) reported using a hormone-based birth control method for pregnancy prevention. Additionally, 51.79% of the participants had heard of or were aware of PrEP as an HIV prevention method before this study (see Table 1 for demographics and sexual behaviors).

**Table 1:** Individual-level variables: demographics, sexual health risk, and HIV prevention behaviors (*n* = 168)

	*n* or *M*	% or *SD*
*Demographics*		
Age (range 18–24)	21.19	1.69
*Education*		
High school	21	12.50
Some college or 2-year degree	78	46.43
Bachelor’s degree or more	67	39.88
*Sexual risk factors*		
Participated in HIV or STI program	32	19.05
Sex in prior 30 days	89	52.98
Sex under the influence	52	30.95
Concurrent sexual partners	18	10.71
Sex with someone met online	62	36.90
Exchange sex	6	3.57
More than 1 sexual partner	97	57.74
Never been tested for STIs	40	23.80
Only tested negative for STIs	105	62.50
Ever tested positive for an STI	23	13.69
Hormone-based birth control during last vaginal sex	87	51.79
Ever heard of PrEP as HIV prevention method	87	51.79
*Outcomes*		
Interest in PrEP	60	35.71
Tested for HIV in prior 3 months	43	25.60
Condom use at last sex	72	42.86

#### Outcome Variables

Among the YBW, 25.60% reported testing for HIV in the prior 3 months, 42.86% reported using a condom during last sex, and 35.71% expressed interest in using PrEP for HIV prevention (see Table 1).

#### Relationship Type

Three types of relationships were examined in this study: family members, friends, and sex partners. Ninety-nine percent of YBW listed at least one friend in their social network (*M* = 6.93; range 0–19), and 89% listed at least one family member (*M* = 3.11; range 0–12). Regarding sex partners, 68% of YBW listed at least one sex partner in their social network, with most women listing one or no sex partners (*M* = 0.95; range 0–4; see Table 2).

**Table 2:** Social network-level descriptive statistics (*n* = 168)

	*n*	%	*M*	*SD*	Range
Total number of SNMs	1808^a^	100	10.76	3.78	5–20
Friend	166	98.81	6.93	3.38	0–19
Family member	149	88.69	3.11	2.18	0–12
Sex partner	114	67.86	0.95	0.86	0–4
Talk about condom use	166	92.22	4.42	3.29	0–16
Friend	137	81.55	3.10	2.99	0–15
Family member	103	61.31	1.21	1.36	0–7
Sex partner	90	53.57	0.66	0.73	0–3
Talk about HIV	104	57.78	1.58	1.78	0–9
Friend	81	48.21	1.03	1.50	0–9
Family member	41	24.40	0.33	0.63	0–3
Sex partner	50	29.76	0.31	0.49	0–2

^a^Based-on total of SNMs across all networks of YBW

#### Communication About Condom Use and HIV Testing

Condom use communication was also high, with 92.22% of YBW speaking to at least one SNM about condom use. Communication about condoms was least likely to occur with family members (60.56%), compared to a friend (81.11%) or a sex partner (77.69%). Communication about HIV testing was lower than condom use. Slightly more than half (57.78%) of YBW reported having spoken with at least one of their SNMs about HIV testing; YBW were least likely to report speaking to a family member about HIV testing (23.33%), followed by a friend (48.33%) or sex partner (61.98%; see Table 2).

### Multivariable Logistic Regression Models

#### Condom Use

In the condom use multivariable logistic regression models (see Table 3), three individual-level variables and two social network-level variables were found to be significant. YBW had decreased odds of reporting condom use during last sex if they had sex in the prior 30 days (AOR 0.15; 95% CI 0.04, 0.61), compared to those who reported not having sex in the prior 30 days. Using hormonal birth control methods also decreased the odds of using condoms during last sex by 74% (AOR 0.26; 95% CI 0.12, 0.58), compared to YBW who did not use hormonal birth control. YBW who have heard about PrEP had 2.47 times the odds of using condoms during last sex (95% CI 1.10, 5.56), when compared to YBW who have not heard of PrEP. At the social network level, YBW who spoke to at least one family member about condom use had 66% decreased odds of using condoms during last sex (AOR 0.34; 95% CI 0.13, 0.87), compared to YBW who did not speak to at least one family member about condom use. However, YBW who spoke to at least one sex partner had 2.99 times the odds of using condoms during their last sexual encounter (95% CI 1.15, 7.77), compared to YBW who did not speak to at least one partner about condom use. There were two variables with a marginally significant (*p* ≤ 0.10) association with condoms use at last sex: having sex with more than one sexual partner (AOR 3.38; 95% CI 0.87, 13.10) and speaking to at least one sex partner about HIV testing (AOR 0.38; 95% CI 0.14, 1.02).

**Table 3:** Multivariable logistic regressions of condom use, HIV testing in prior 3 months, and Interest in PrEP (*n* = 168)

	Condom use		HIV testing in prior 3 months		Interest in PrEP	
	*AOR*	95% CI	*AOR*	95% CI	*AOR*	95% CI
*Demographics*						
Age	0.91	0.68, 1.22	0.81	0.58, 1.13	1.11	0.82, 1.50
Education^a^						
Some college or two-year degree	1.85	0.58, 5.88	1.43	0.38, 5.45	1.19	0.36, 3.92
Bachelor’s degree or more	1.92	0.48, 7.66	1.44	0.31, 6.83	0.63	0.15, 2.62
*Sexual risk factors*						
Participated in HIV STI program	0.72	0.28, 1.90	1.19	0.41, 3.44	1.54	0.59, 4.01
Sex in last 30 days	0.15**	0.04, 0.61	0.93	0.21, 4.20	1.12	0.28, 4.45
Hormonal birth control	0.26**	0.12, 0.58	0.59	0.24, 1.46	1.34	0.58, 3.11
Sex under the influence	1.49	0.66, 3.35	0.62	0.25, 1.54	0.68	0.30, 1.54
Concurrent sexual partners	0.66	0.19, 2.32	1.28	0.31, 5.31	1.11	0.32, 3.81
Sex with someone met online	1.61	0.71, 3.66	0.84	0.32, 2.21	1.89	0.84, 4.27
More than 1 sexual partner	3.38^	0.87, 13.10	1.28	0.30, 5.54	1.63	0.41, 6.43
Tested negative for STIs^b^	0.58	0.22, 1.50	2.01	0.59, 6.77	3.03*	1.05, 8.73
Ever tested positive for an STI^b^	0.44	0.11, 1.80	1.48	0.26, 8.44	7.92**	1.71, 36.66
Heard of PrEP	2.47*	1.10, 5.56	2.25^	0.88, 5.72	0.52	0.22, 1.18
*Outcomes*						
Interested in PrEP	1.24	0.55, 2.79	0.33*	0.13, 0.85	–	–
Tested for HIV in prior 3 months	1.55	0.66, 3.66	–	–	0.37*	0.14, 0.93
Condom use	–	–	1.59	0.66, 3.86	1.23	0.54, 2.81
*Social network variables*						
Talk about condom use						
Family member	0.34*	0.13, 0.87	1.52	0.49, 4.71	1.35	0.51, 3.57
Friend	1.81	0.58, 5.65	0.28	0.08, 1.04	2.32	0.65, 8.20
Sex partner	2.99*	1.15, 7.77	0.19*	0.06, 0.63	0.30*	0.11, 0.78
Talk about HIV testing						
Family member	1.49	0.46, 4.84	1.94	0.55, 6.92	0.49	0.15, 1.63
Friend	0.88	0.35, 2.23	3.21^	0.98, 10.47	2.58^	0.98, 6.80
Sex partner	0.38^	0.14, 1.02	3.97*	1.25, 12.66	1.94	0.69, 5.46
*R*^2^	.20	.20	.19			

^a^Reference category was high school

^b^Reference category was never been tested for STIs

^*p* < .10; *p < .05; **p < .01; ***p < .001

#### HIV Testing

One individual-level and two social network-level variables were found to be significantly associated with being tested for HIV in prior 3 months (see Table 3). YBW who reported being interested in PrEP use as a HIV prevention method had 67% decreased odds of being tested for HIV in the prior 3 months (AOR 0.33; 95% CI 0.13, 0.85), compared to YBW who were not interested in PrEP use. At the social network level, YBW who spoke to at least one sex partner about condom use had 81% decreased odds of being tested for HIV in the prior 3 months (AOR 0.19; 95% CI 0.06, 0.90), compared to YBW who did not speak to a sex partner about condom use; and YBW who spoke to at least one sex partner about HIV testing had 3.97 times the odds of being tested for HIV in the prior 3 months (95% CI 1.25, 12.66), compared to YBW who did not report speaking to at least one sex partner about HIV testing. There were two variables with a marginally significant (*p* ≤ 0.10) association with being tested for HIV in the prior 3 months: having heard of PrEP and speaking to at least one friend about HIV testing. YBW who reported being aware of PrEP as a method of HIV prevention had marginally significant increased odds of being tested for HIV in the prior 3 months (AOR 2.25; 95% CI 0.88, 5.72). YBW who reported speaking to at least one friend about HIV testing had marginally significant increased odds of being tested for HIV in the prior 3 months (AOR 3.21; 95% CI 0.98, 10.47).

#### Interest in PrEP

Three individual level variables and one social network level variable were associated with interest in using PrEP as an HIV prevention method (see Table 3). YBW who reported testing for HIV in the prior 3 months had 63% decreased odds of being interested in PrEP use for HIV prevention (AOR 0.37; 95% CI 0.14, 0.93), compared to those who did not report testing for HIV in the prior 3 months. YBW who ever tested negative for STI had a 3.03 increased odds of being interested in PrEP (95% CI 1.05, 8.73), compared to YBW who never tested for any STIs (95% CI 1.71, 36.66), and YBW who ever tested positive for an STI had 7.92 increased odds of being interested in PrEP compared to YBW who never tested for any STIs. YBW who spoke to at least one sex partner about condom use had 70% decreased odds of being interested in PrEP (AOR 0.30; 95% CI 0.11, 0.78), compared to those who did not report speaking to at least one sex part about condom use. Regarding marginally significant relationships (*p* ≤ 0.10), YBW who reported talking to at least one friend about HIV testing had increased odds of being interested in PrEP use (AOR 2.58; 95% CI 0.98, 6.80).

## Discussion

With the increase in STIs and HIV among young adults, particularly YBW, a concerted effort focusing on contextually relevant strategies is paramount. This study sought to examine how individual-level factors and social network-level factors were associated with condom use, HIV testing, and interest in use of PrEP among YBW. Study findings reveal that factors at both the individual and social network levels were associated with condom use, HIV testing, and interest in PrEP among YBW.

Results from this study suggest relatively high levels of condom use negotiation skills among YBW and their sex partners, because many YBW reported speaking to at least one sex partner about condom use, and speaking with a sex partner about condom use was significant associated with condom use during last sexual encounter. This is promising because HIV prevention interventions for Black women have focused on increasing condom negotiation between Black women and their sex partners (Chandler et al., 2016; Javier et al., 2018). Consistent with the literature, although YBW appear to be taking active and assertive roles in condom use negotiation and discussions with their partners, they are still reporting inconsistent condom use (McLaurin-Jones et al., 2016). One reported reason for the low condom use, despite the increase in condom use conversations, is the use of birth control (McLaurin-Jones et al., 2016). Our finding reveal that YBW were significantly less likely to use a condom during their last sexual encounter if they reported using hormonal birth control (e.g., pill, IUD, patch, Nexplanon). This finding aligns with prior research that found YBW are more concerned about avoiding pregnancy then preventing STIs and HIV and see condoms as a pregnancy prevention method and not an HIV prevention method (Anakaraonye et al., 2019). Thus, if YBW are using a hormonal birth control method, condoms may not be considered necessary. Qualitative studies that examine YBW and their partners’ birth control and condom use communication and decision making are needed to better understand the nuance of these conversations, including the context of the conversations, how these conversations occur, and how condom use decisions are made when it comes to HIV and pregnancy prevention. In addition to understanding the context of these conversations, messaging around the use of condoms for YBW and their partners should be tailored to focus on condom use as being not only birth control, but also a means of STI and HIV prevention.

Interestingly, YBW who reported speaking with their sex partners about condom use and those who tested for HIV in the prior three months were less likely to be interested in PrEP. We speculate that such disinterest may be connected to self-perceived risk. Recent studies have found that YBW with increased self-perceived HIV risk had an increased interest in PrEP (Park et al., 2019; Sales & Sheth, 2019; Sewell et al., 2020), as did Black women with a recent STI (Hirschorn et al., 2020), which was supported by our findings with YBW who ever tested for STIs (both positive and negative results) having a higher odds of being interested in PrEP when compared to those never tested for an STI. Black women have indicated that conversations with sex partners about condom use, along with trust in their partners, alleviates some of the stress around perceived risk of HIV (Caldwell & Mathews, 2015). Thus, condom use conversations with sex partners and testing for HIV in the prior three months may have decreased YBW’s self-perceived HIV risk and resulted in lower interest in PrEP in this sample, particularly if they were more likely to use condoms during sex.

It is also notable that talking with sex partners about HIV testing was significantly associated with being tested for HIV. This finding aligns with a recent study of young Black women and girls (ages 13–25) that found that engaging in sexual health conversations and reporting higher levels of comfort discussing HIV with a partner were associated with being tested for HIV (Boyd et al., 2018). As our results stem from cross-sectional data, we cannot determine if YBW are talking with their sex partners about HIV testing then getting tested for HIV, or if YBW are talking with their sex partners about HIV testing because they have been tested for HIV. But like conversations around condom use, what is most important is these conversations around HIV testing are happening. However, to understand the impact of HIV testing communication and HIV testing behaviors time order, longitudinal studies are needed.

Notably, there are some complex relationships between variables, heard of PrEP, interest in PrEP, and being tested for HIV in the prior 3 months. YBW who reported ever hearing about PrEP had higher odds of being tested for HIV in the prior 3 months. However, YBW who were tested for HIV in the prior 3 months were significantly less likely to be interested in PrEP use. There was no significant relationship between being aware of PrEP and interest in PrEP. The lack of association between PrEP awareness and interest in PrEP and the negative association between HIV testing and interest in PrEP could be due to inadequate understanding of the benefits of using PrEP as a method for HIV prevention. The use of this HIV medication is effective in reducing HIV incidence rates and has been used to help overcome challenges in the uptake of other preventive strategies (Montgomery et al., 2019; van der Straten et al., 2012). However, in this sample, we speculate that the lack of a direct association between PrEP awareness and interest in PrEP and negative association between HIV testing and interest in PrEP can also highlight a misunderstanding of who can benefit from the use of PrEP (e.g., YBW participating in unprotected sex with partners whose status is unknown). Given that the sample was found to have inconsistent condom use, it is important to enhance interest in PrEP to further mitigate HIV transmission. Utilization of family planning and youth-friendly clinics and technological interventions such as mobile applications may facilitate uptake of PrEP in this population, as seen in similar populations globally (Celum et al., 2019). Furthermore, research is needed to better understand for whom YBW perceive PrEP is intended and YBW’s perceptions of the risk level that warrants PrEP use, so misconceptions and misinformation can be better addressed in HIV preventive interventions.

### Limitations

Although this study revealed notable findings, there are several limitations to consider. This study was cross-sectional, which limited the ability to make causal links between HIV prevention behaviors and individual- and social network-level factors. Data were self-reported, which may increase social desirability bias. Additionally, this study was respondent driven, thus this sample may have homogeneous behaviors at the individual- and social network-levels and statistical inferences must be taken with precaution. Outcome models were not able to adjusted for potential clustering resulting from RDS, due to the lack of link data for referral and seed participants. Due to the smaller sample size include in these models (*n* = 168) and the number of variables examined in these models, the accrued odds ratios in these models may not be as precise, as indicated by the wider confidence intervals. Although many of the findings are supported by the literature, future studies should examine similar factors in a larger sample of YBW to increase the confidence in findings and to allow for the inclusion of additional sociodemographic factors, such as household income, sexual orientation, employment status, gender of sex partner and relationship status. Furthermore, this study did not assess the content or tone of conversations about condom use or HIV, which remains a gap in our understanding of this topic.

### Implications and Conclusion

Finding from this study have the potential to improve interventions geared toward YBW. This study highlighted that both individual- and social network-level factors should be accounted for when addressing sexual health promotion and HIV prevention among YBW. Specifically, these results reveal that speaking with sex partners about condom use was the only factor significantly associated with HIV prevention behaviors; thus, we must consider leveraging these sexual relationships in HIV interventions for YBW. Although this study did not examine sexual health communication injunctive norms regarding condom use, STI testing, or HIV testing, findings from this study support previous research that has underscored the importance of any sexual health communication among target populations and their SNMs (Barman-Adhakari et al., 2018; Cederbaum et al., 2017; Craddock, 2020; Craddock et al., 2016, 2020; Widman et al., 2016). To further understand the relationship between sexual health communication and HIV prevention behaviors and better tailor HIV interventions for YBW, future research must consider the context in which these conversations occur (e.g., beginning of sexual relationships, before sex, or after testing for STIs), the injunctive norms associated with HIV prevention behaviors, and why YBW do or do not use HIV prevention methods.

## Data Availability

Data are available by request.

## Code Availability

SAS codes are available by request.
